# Item Analysis of Multiple-Choice Question (MCQ)-Based Exam Efficiency Among Postgraduate Pediatric Medical Students: An Observational, Cross-Sectional Study From Saudi Arabia

**DOI:** 10.7759/cureus.69151

**Published:** 2024-09-11

**Authors:** Khaled A Shahat

**Affiliations:** 1 Pediatric and Adolescent Medicine, Alrayan National College of Medicine, Madinah, SAU; 2 Pediatric and Adolescent Medicine, College of Medicine, Taibah University, Madinah, SAU

**Keywords:** discrimination index, difficulty index, reliability, validity, assessment in education

## Abstract

Background: Several modalities of written examination have been employed in medical education, with multiple-choice questions (MCQs) being the most frequently used and preferred format. This underlines the need to regularly assess and monitor the quality of MCQs in medical exams. Such assessment of MCQs helps to ensure that these exams are well‑designed and adequately powered to evaluate students’ performance. Hence, the current study assessed the efficiency of an MCQ-based examination, in a cohort of pediatric post-graduate students in Saudi Arabia.

Methods: This observational, cross-sectional study examined the efficiency of MCQs in terms of their validity, reliability, difficulty index (DFI), discrimination index (DI), and distractor efficiency (DE). The exam consisted of a total of 48 MCQs, 144 distractors, a total score of 48, and no negative marking.

Results: The reliability index of 0.76 showed the consistency and reproducibility of the exam results. The exam had a DFI of 69.77%, indicating an overall moderate level of difficulty. The exam had a balanced mix of 23 easy (47.9%), 20 (41.7%) moderately difficult, and five (10.4%) tough questions. Twenty (41.6%) items had a DI of ≥0.3, indicating good discrimination of high and low performers, while the remaining 28 MCQs (58.3%) had a lower DI of ≤0.19, implying poor discriminative ability. The DE was 81.25%, indicating that the majority of distractors in the exam were functional.

Conclusion: To the best of the author’s knowledge, this is the first study among post-graduate pediatric students from Saudi Arabia, to present the results of item analysis of an MCQ-based exam. The study highlights the importance of optimizing the quality of MCQs by following established guidelines, to make MCQ-based clinical assessments more effective. It reiterates the importance of a reasonable DFI well aligned with students’ knowledge levels, maximum distractor functionality, and an impactful DI, in developing high-quality MCQs.

## Introduction

Written assessments are vital in education as they measure students' capacity to attain learning objectives and bridge the gap between teaching and understanding [1]. In addition to providing feedback on students’ performance, such assessment allows evidence-based monitoring of students’ progress and learning outcomes [2]. The formative type of assessment helps students to identify strengths and potential areas of improvement, while summative assessment provides a tangible evaluation of students’ progress [3].

Various assessment methods have been used for different evaluation goals, including multiple-choice questions (MCQs), objective structured clinical examination (OSCE), descriptive papers, and medical essays. [4]. Among these methods of assessment, MCQs represent the most widely employed and preferred tool [5]. However, an MCQ-based examination achieves the desired efficiency of evaluation, only when the MCQs are of an optimum quality and are well-structured. Routinely, MCQs are composed of three parts: the stem, the key, and the distractors; with the key representing the correct answer and the distractors serving as incorrect options. To avoid placement bias, correct answers should be evenly distributed throughout the test. Moreover, effective distractors should be chosen from among students with poor performance. The efficiency of MCQs is further enhanced by ensuring that the key and distractors are similar in length, style, and grammatical form [6-8].

For a comprehensive assessment of MCQ quality, specific parameters have been identified such as validity, reliability, standard error of measurement, difficulty index (DFI), discrimination index (DI), and distractor efficiency (DE). The validity of test scores as an assessment tool has been proven by evidence and theory [9,10]. It can be adjudged on the basis of the test content and its internal structure [11]. The test's content should be in congruence with the intended objectives of the test, it should adequately represent the key areas of assessment on a given topic [12]. Internal structure pertains to the statistical and psychometric characteristics of the test, such as item difficulty, item discrimination, reliability, and standard error of measurement [13]. Reliability refers to the consistency or repeatability of measurement scores and represents one of the essential criteria for validity [14].

There is a dearth of data from Saudi Arabia, regarding the efficiency of MCQs as an exam tool, especially among students from the post-graduate pediatric specialty. Hence, the current study was designed and conducted as an attempt to fill this evidence gap, by performing an item analysis of an MCQ-based test, administered to a cohort of Saudi students in the post-graduate pediatric program.

## Materials and methods

This was a prospective, cross-sectional study conducted during the academic year 2021-2022 at Taibah University, Madina, Saudi Arabia. During the study, the “MCQ End Block Exam for the Integrated Specialty Clinical Practice-1 (Child Health) Course” was administered to 46 medical students. The exam consisted of 48 MCQs with a total score of 48 and no negative marking.

Item analysis was conducted to assess the quality of the MCQs. This analysis involved determining the reliability, item difficulty, item discrimination, standard error of measurement, and DE. Reliability was measured using the Cronbach's alpha coefficient. The standard error of measurement was calculated using a formula that considers the standard deviation of scores and the reliability coefficient [15].

The item DFI, rated between 0% and 100%, is a measure of the proportion of students who answered an exam item correctly. The index's proximity to 100% suggests easy questions, while closer to 0% represents difficult questions. Ideally, exam items should have a balanced DFI, where most items fall between the 30% and 70% range, to adequately challenge students without overwhelming or discouraging them. Items having DFI below 30% and above 70% are considered difficult and easy items, respectively [15].

Item DI is a statistical method that assesses the extent to which each item distinguishes between students who perform highly and those who perform poorly in an exam. The DI, ranging from 0 to 1, assesses a question's capability to differentiate high-performing and low-performing students. A higher index score indicates an effective question, while a lower score implies a poor question. The DI of a good item ought to be 0.3 or greater. Item discrimination was calculated by comparing the scores of the top and bottom-performing students, with a point‑biserial correlation coefficient calculated for each question [15].

Distractor analysis was performed by determining the proportion of students who selected each incorrect answer choice for each question. Distractors not chosen by any students were deemed as non-functional. The number of non-functional and functional distractors was incorporated as a key parameter in the analysis of DE. The mean of DE scores of all 48 questions was calculated to find out the overall DE of the examination. DE for a particular MCQ is calculated based on the number of non-functional distractors: A question without any non-functional distractor amounts to DE of 100%, two non-functional distractors indicate a DE of 66.66%, three indicate a DE of 33.33%, and four indicate a DE of 0. [15].

## Results

Table 1 provides an overview of the scores obtained for key parameters of exam efficiency. The median score of 34.00 indicates that half of the students did perform better than this score, while the other half scored below it. The average score of 33.72 indicates a mildly negatively skewed distribution of scores. The standard error of measurement of 2.80 indicates that the exam scores seem to be fairly reliable, though with a moderate level of measurement error. The standard deviation of 5.72 conveys a moderate degree of variation in the students' scores. The minimum score of 22.00 and the maximum score of 44.00 demonstrate a wide range of performance among the students. The exam's total difficulty was calculated to be 69.77%, indicating a moderately difficult level, while the reliability index (KR20) of 0.76, indicates the exam's acceptable consistency and reproducibility.

**Table 1:** Summary of key parameters of exam efficiency

Key parameters of exam efficiency	Results
Exam median score	34.00
Exam average score (Mean)	33.72
Standard error of measurement	2.80
Standard deviation (SD)	5.72
Minimum score	22.00
Maximum score	44.00
Total difficulty	69.77%
Reliability index (KR20)	0.76

The DFI varied from 0% to 100%, signifying a broad range of question difficulty. The exam had a balanced mix of 23 easy (47.9%), 20 (41.7%) moderately difficult, and five (10.4%) tough questions. For instance, question 11 was incorrectly answered by all students, signifying that the question was either too difficult or might have been inappropriately formulated. Likewise, question 19 was answered correctly by merely 17.4% students, indicating a high difficulty level. On the contrary, 19 other questions had a low difficulty level with a DFI greater than 80%. For instance, question 1 was answered correctly by 95.7% students. Table 2 presents the DFI for each question or item.

**Table 2:** Item analysis of DFI and DI in the MCQ-based examination DFI – Difficulty Index; DI – Discrimination Index; MCQ – Multiple Choice Question

Question	Difficulty index	Discrimination index
1	95%	0
2	95%	0.12
3	82%	0.06
4	30%	0.45
5	84%	0.12
6	63%	0.3
7	73%	0.3
8	65%	0.18
9	54%	0.51
10	60%	0.63
11	0%	0
12	97%	0.06
13	60%	0
14	19%	0.06
15	80%	-0.12
16	71%	0.45
17	69%	0.18
18	67%	0.39
19	17.4%	0.45
20	80%	0.39
21	86%	0.12
22	84%	0.18
23	93%	0.06
24	95%	0.06r
25	73%	0.12
26	67%	0.3
27	89%	0.12r
28	91%	0.24
29	69%	0.3
30	78%	0.18
31	89%	0.06
32	93%	0.18
33	84%	0.12
34	80%	0.18
35	82%	0.39
36	26%	0.45
37	32%	0.39
38	21%	0.06
39	69%	0.39
40	23%	0.06
41	63%	0.57
42	93%	0
43	78%	0
44	82%	0.39
45	43%	0.63
46	30%	0
47	54%	0.39
48	84%	0.3

In this test, the DI ranged from -0.12 to 0.63. Twenty (41.6%) items had a DI of ≥0.3 indicating good discrimination of high and low performers, while the remaining 28 MCQs (58.3%) had a lower DI of ≤0.19, implying poor discriminative ability of these questions. The test had one question or item with a negative DI, implying that the majority of high performers chose the incorrect option for this item. Six other items showed a DI of zero; indicating that these items were completely ineffective at distinguishing between the two groups of students. Table 2 presents the DI for each question or item.

The overall DE of the test was 81.25%, indicating a fairly good level of functional distractors. The large majority of items (31 out of 48 (64.58%)) had no non-functional distractors. Sixteen (33.3%) questions had at least one unused distractor, indicating suboptimal use of distractors in these questions. Six other items had their distractors selected more frequently than the correct answer. This implies that students may have been misled into choosing incorrect answers, despite possessing the required knowledge, to select the right answers. However, Table 3 summarizes the analysis of DE in the examination.

**Table 3:** Analysis of distractor efficiency

Distractor status	Distractor efficiency	No of items out of 48 (%)
Items with 0 non-functional distractor	100%	31 (64.58)
Items with 1 non-functional distractor	66.66%	8 (16.67)
Items with 2 non-functional distractors	33.33%	8 (16.67)
Items with 3 non-functional distractors	0	1 (2.08)
Total distractor efficiency	81.25%	

## Discussion

In the current study, the mean score of test results was 33.72, out of a total mark of 48. In the author’s opinion, this is a fairly satisfactory mean score which signifies that the majority of students had a good understanding of the subject matter and the MCQ test facilitated them to successfully apply their knowledge. Reasonably good performance by students in this MCQ-based test which had a moderate difficulty level is an encouraging outcome and favors the use of the MCQ-based exam format. The test’s reliability index of 0.76 also falls within the recommended range of 0 to 1, with ≥0.8 being ideal. Hence, a reliability value of 0.76 strongly points towards the good consistency and reproducibility of the test results.

The exam had a DFI of 69.77%. This is within the widely recommended and accepted DFI range of 30%-70% [15]. However, as the DFI of this test is almost nearing the upper limit of the accepted range, it can be inferred that the test had a moderate level of difficulty. The exam had a balanced mix of 23 easy (47.9%), 20 (41.7%) moderately difficult, and five (10.4%) tough questions. Previous similar studies have also demonstrated a DFI ranging between 30% and 70%. These studies showed a mix of easy as well as tough questions, with the majority of items having an average difficulty level. This trend of previous studies seems similar to the difficulty pattern seen in our study [16-20]. In the current study, the questions were adequately structured and the test’s DFI seems well-attuned to students’ knowledge levels. The author believes that such tests with a balanced mix of easy and difficult questions provide a fair estimation of students’ preparedness and knowledge, without making students too over-confident or getting them discouraged.

In this test, the DI ranged from -0.12 to 0.63, with 20 (41.6%) questions having a DI ≥0.3. This implies that these 20 items were adequately effective at differentiating between high-performing and low-performing students [15]. Among these 20 questions, a total of 15 (31.2%) questions showed excellent discrimination. However, another 20 (41.6%) items had a DI <0.19, implying poor discriminatory ability. As per guidelines, such items should either be rejected and replaced by better-formulated items or be improvised to achieve a better DI. One (2.08%) item showed a negative DI which means that the majority of high performers chose the incorrect option for this item. Most probably this item was inappropriately formulated, often misleading towards the wrong answer. Six (12.5%) other items showed a DI of zero; indicating that these items were completely ineffective at distinguishing between low versus high-performing students. Overall, as per the item analysis, the DI of the test showed a scope for improvement. This emphasizes the need to formulate MCQs with greater discriminative power for designing efficient examinations. Pande et al. conducted an item analysis of 240 MCQ items, across physiology tests conducted for 100 students, over four academic years. Their study showed a DI ranging from -0.54 to 0.8, with a mean of 0.3. Twenty-nine percent of the items in this study showed excellent discrimination, 46% had good-to-acceptable and 21% items had poor discriminative ability. Four percent of the items also showed negative discrimination. The study by Pande et al. showed a better discrimination profile on item analysis, with a remarkably lower percentage of poor discriminators, as compared to the current study [21]. However, Pande et al. did show a mix of excellent, good, acceptable, poor, and negative discrimination; a feature that was also seen in the current study. The study by Kumar et al. involved item analysis on 90 MCQs, of these 72 (80%) had an excellent DI. This is an exemplary finding that indicates excellent discriminative ability of the large majority of items in the test [22]. In sharp contrast to this, Bhattacherjee et al. published the results of an item analysis on 60 medical MCQs and found that >70% items had a poor DI [23]. Perhaps, the published literature seems to present a variable trend regarding discrimination indices of MCQ-based tests. In the author’s opinion, DI can be impacted by the complexity of the subject matter, the significance of the exam (high versus low stakes), the difficulty level of the test, and the overall knowledge level and preparedness of students.

The author believes that one of the key objectives of any examination is to identify poor performers, elicit the causes of low performance, and plan remedial measures to address the same. A good DI assumes paramount importance in this context, as it helps identify poor performers who would need better academic focus, more intensive coaching, and greater faculty support. The author also believes that the high‑performers identified through the DI; can be encouraged to scale up further academically, and also help their poor-performing colleagues via peer-to-peer learning models.

The study had an appreciable DE of 81.25%. This proves that the large majority of distractors in the test were functional and optimally utilized by students. However, the test did have a few distractors that remained unutilized, under-utilized, or that misled students into selecting the wrong option. Past evidence has shown that the use of poorly designed and misleading distractors can significantly impact the accuracy and reliability of the exam, leading to potential distortions in interpreting students’ performance levels. Hence, it is essential to develop distractors that are derived from theoretical or practical misconceptions among students. It is also important to meticulously select only those distractors that seem plausible but are yet incorrect. These steps can help enhance distractor functionality [24].

Items with a higher number of functional distractors help in raising the difficulty and discrimination level of the examination. Items with four or five options are most widely used in medical examinations. However, a growing body of research supports using two or three options in MCQs because this reduces the total number of distractors, improves the overall quality of distractors, reduces the overall time needed to construct the test, and is largely preferred by students versus the “four or five option” model. Faculty members often find it challenging to develop efficient distractors; hence, item analysis can be used to detect and delete non-functional distractors from MCQs that have been used previously [25].

Automated tools such as software-generated MCQs have been attempted with a fair degree of success. Artificial intelligence, machine learning, and deep learning models have shown promise in the automated generation of MCQs based on information fed through large pools of previous data [26,27]. In the author’s opinion, such automation has the potential to make the MCQ generation process smoother, less laborious, faster, and with minimum possibility of human errors [28]. However, the practical feasibility of such technological upgrades, at a large-scale level, needs to be revisited given their cost and infrastructural implications.

Nevertheless, the current study had its own set of limitations. This was a single-center study, and the results might not be comparable to those of a large-scale, multicentric study, which often provides more comprehensive and robust evidence. Besides, the item analysis presented in this study was based on a single post-graduate pediatric examination. Future studies involving item analyses conducted on a series of such examinations could provide long-term data, with greater implications for improvement.

## Conclusions

To the best of the author’s knowledge, this is the first study among post-graduate pediatric students from Saudi Arabia, to present the results of item analysis of an MCQ-based exam. The present study highlights the importance of optimizing and upgrading the quality of MCQs by following established guidelines, to make MCQ-based clinical assessments more effective and meaningful. It reiterates the importance of a reasonable DFI, which is well aligned with students’ knowledge levels, maximum distractor functionality, and an impactful DI. The study points toward exploring the relationship between item quality and students’ performance in exams, as a potential area of future research. Similar future studies can be designed to assess the quality of MCQs in different curricula of medical education.
